# Incisional Hernia in a 12-mm Nonbladed Trocar Site Following Laparoscopic Nephrectomy

**DOI:** 10.1100/tsw.2006.372

**Published:** 2006-03-26

**Authors:** Erik J. Kouba, J. Slade Hubbard, Eric Wallen, Raj S. Pruthi

**Affiliations:** Division of Urologic Surgery, The University of North Carolina at Chapel Hill, NC, USA

**Keywords:** laparoscopy, nephrectomy, hernia, port, complications

## Abstract

Non-bladed trocars and radially dilating systems are considered less traumatic to the abdominal wall because they do not incise the fascia itself. Since the fascia is not cut, it has believed that the fascia closes by itself. Consequently, several authors have suggested that closure of the abdominal fascia may be unnecessary when such non-bladed laparoscopic trocars are used. We report of a case in which a port site hernia was diagnosed at the site of a 12 mm non-bladed trocar 11 days after laparoscopic nephrectomy. Although it may be true that in many cases port site closure is unnecessary and does not result in bowel herniation, this case along with a prior report serve as important reminders that port site hernias are possible even in the use of non-bladed or radial dilating systems, and that there exists a number of potential variables that may predispose to herniation and consequently the ability to predict such events in individual patients remains uncertain. As such, we recommend closing 10 mm or larger port sites irrespective of trocar design.

## INTRODUCTION

Nonbladed trocars, radially dilating systems, and conical blunt devices are considered less traumatic to the abdominal wall because they do not incise the fascia itself. Rather, the tissue fibers are arrayed in a criss-cross fashion as the trocar stretches the fibers of the fascia. Since the fascia is not cut, it is believed that the fascia closes by itself. Moreover, it has been postulated that the misalignment of the fascial defects may inhibit herniation[1]. Consequently, several authors have suggested that closure of the abdominal fascia may be unnecessary when such nonbladed, laparoscopic trocars are used[2,3]. We report a case in which a port site hernia was diagnosed at the site of a 12-mm, nonbladed trocar 11 days after laparoscopic nephrectomy.

## CASE REPORT

A 63-year-old female underwent a hand-assisted, left laparoscopic, radical nephroureterectomy at an outside institution for transitional cell carcinoma of the left renal pelvis. The procedure itself was deemed uneventful. A 7-cm incision for the hand-assist port was made just cephalad to the umbilicus, and two 12-mm nonbladed ports were placed in the left upper quadrant and left lower quadrant (Ethicon Endosurgical Inc, Cincinnati, OH). Of note, there was not fascial closure at the 12-mm port site during the original operation due to use of the nonbladed trocars. This was confirmed by the operative report and through personal correspondence with the original surgeon.

The patient had an unremarkable hospital stay and was discharged on the fifth postoperative day. Six days after discharge (postoperative day number 11) she presented to our facility with a 5-day history of persistent nausea, vomiting, and left lower quadrant pain. Physical exam and abdominal computerized tomography (CT) revealed findings consistent with possible bowel obstruction secondary to port site hernia in the left lower quadrant (Fig. 1). The patient underwent an exploratory laparotomy through the prior hand port site. On exploration, a loop of incarcerated small bowel was noted at the level of the interior abdominal

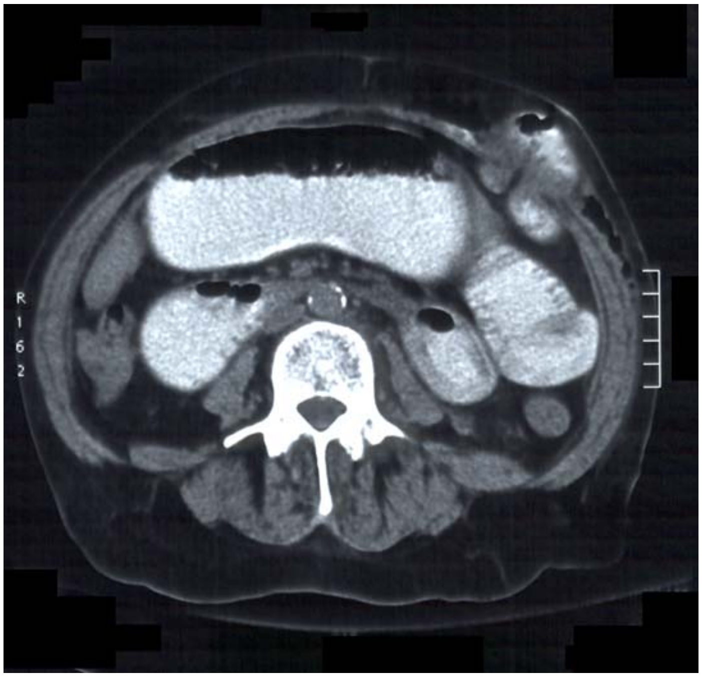

**FIGURE 1.** Abdominal CT revealing herniation of small bowel at the location of the left lower 12-mm port site.

wall fascia through the 12-mm port site in the left lower quadrant (Fig. 2). The bowel was reduced, a 10-cm segment resected (due to areas of bowel ischemia at site of incarceration), and the small bowel was reanastomosed. Fascial closure of both 12-mm port sites and the midline incision was performed. The patient had an unremarkable postoperative course and was discharged on postoperative day number 5.

## DISCUSSION

This is the second reported case of a symptomatic, incisional port site hernia using 10-mm or larger nonbladed laparoscopic trocars without fascial closure[4]. The sequelae of bowel herniation through port sites are significant and may include emergent reoperation, as in this case, or even more critical outcomes.

Several investigators have examined the incidence of incisional hernias after the use of nonbladed laparoscopic trocars without fascial closure. In a series of 70 patients undergoing laparoscopic live donor nephrectomy, Siqueira et al. have reported that no patients had developed symptomatic hernias in which the fascia of nonbladed 12-mm ports was not closed[5]. Additionally, in a prospective study of 244 patients comparing 10-mm port-site fascial closure vs. nonclosure, Bhoyrul et al. reported that no patient developed an incisional hernia after elective laparoscopic procedures in either cohort[6]. In another study of fascial nonclosure, Liu et al. observed no incisional hernias in 70 patients after laparoscopic surgery using nonbladed trocars[1].

The rationale for nonclosure of blunt trocar sites is derived from several animal studies that have shown that blunt trocar fascial defects were smaller in area, shorter in length, shorter in width, and had less destruction of fascial tissue compared to bladed trocars. Furthermore, separation (rather than transection) of musculature has been observed with blunt trocar insertion, and this separation generally reapposes after trocar removal[7,8].

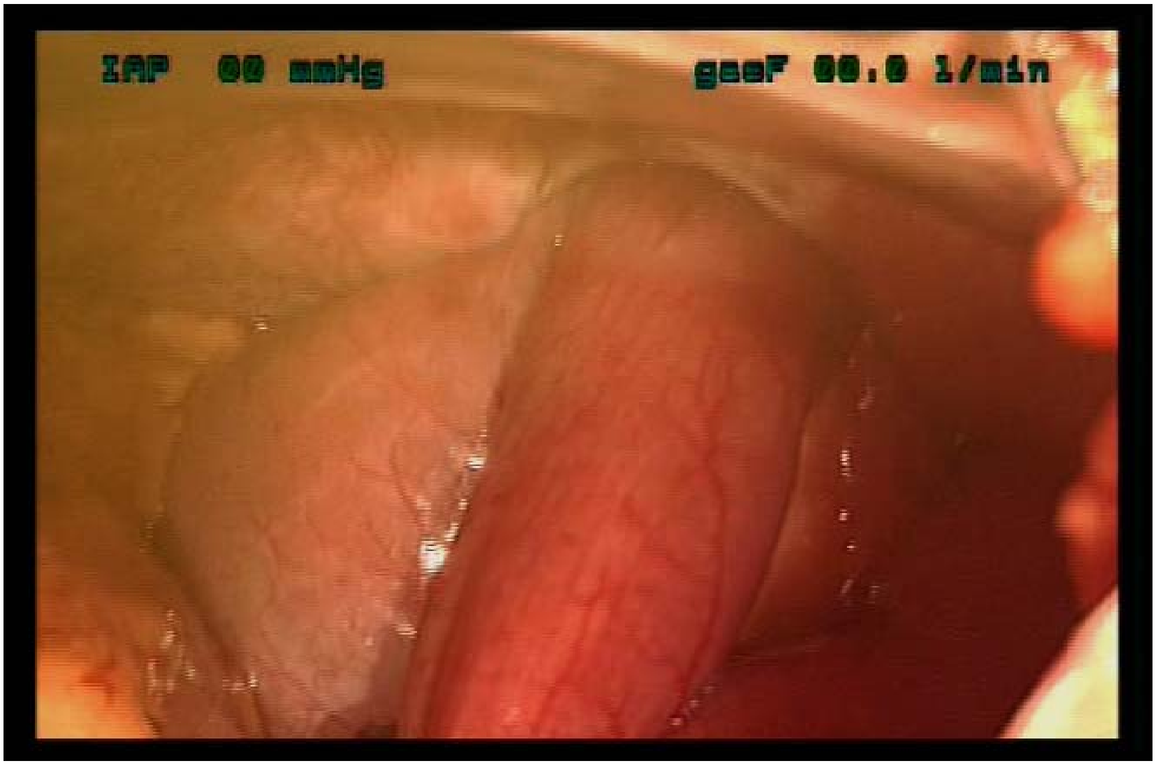

**FIGURE 2.** Intraoperative picture showing the loop of ileum herniated through the left lower 12-mm port site. Note the dilated proximal limb and the decompressed distal limb at the point of herniation.

However, such preclinical study does not necessarily support the elimination of fascial closure in noncutting trocars due to several variables that may be present during nonexperimental conditions. First, the use of cannulas made of different materials of construction (i.e., thick plastic as compared to thin steel) may add several millimeters to the outside diameter of the cannula (despite a similar internal diameter), and this may result in a larger radius of tissue damage. Second, factors such as the angle of insertion, repeated movements of the cannula while changing instruments, the use of large forces to insert the trocar, and multiple thrusting attempts during entry can impact fascial damage[7,9]. Third, forced dilation of the fascial layer during specimen removal may increase port site damage and might be involved with occurrence of trocar site hernias[10,11]. Last, patient comorbidities such as obesity, infection, steroids use, and diabetes mellitus can also influence the incidence of trocar site hernias[10,11].

In conclusion, although it may be true that in many cases port site closure is unnecessary and does not result in bowel herniation, this case along with the prior report in a transplant donor serve as important reminders that port site hernias are possible even in the use of nonbladed or radial dilating systems[4]. There exist a number of potential variables that may predispose to herniation and consequently the ability to predict such events in individual patients remains uncertain. Moreover, port site closure techniques such as the Endoclose suture and the Carter-Thomason device, among others, have allowed secure and efficient port closure[12]. As such, we recommend closing 10-mm or larger port sites irrespective of trocar design.
